# Control of *n*-Butanol Induced Lipidome Adaptations in *E. coli*

**DOI:** 10.3390/metabo11050286

**Published:** 2021-04-29

**Authors:** Aike Jeucken, Miaomiao Zhou, Marc M. S. M. Wösten, Jos F. Brouwers

**Affiliations:** 1 Membrane Enzymology, Groningen Biomolecular and Biotechnology Institute (GBB), University of Groningen, 9747 AG Groningen, The Netherlands; a.jeucken@rug.nl; 2 Research Group Analysis Techniques in the Life Sciences, School of Life Sciences and Environmental Technology ATGM, Avans University of Applied Sciences, 4818 AJ Breda, The Netherlands; m.zhou1@avans.nl; 3 Infection Biology, Department of Biomolecular Health Sciences, Utrecht University, 3584 CL Utrecht, The Netherlands; m.wosten@uu.nl; 4 Center for Molecular Medicine, University Medical Center Utrecht, 3584 CX Utrecht, The Netherlands

**Keywords:** *Escherichia coli*, butanol, lipidomics, mass-spectrometry, phospholipid

## Abstract

The versatile compound *n*-butanol is one of the most promising biofuels for use in existing internal combustion engines, contributing to a smooth transition towards a clean energy society. Furthermore, *n*-butanol is a valuable resource to produce more complex molecules such as bioplastics. Microbial production of *n*-butanol from waste materials is hampered by the biotoxicity of *n*-butanol as it interferes with the proper functioning of lipid membranes. In this study we perform a large-scale investigation of the complete lipid-related enzyme machinery and its response to exposure to a sublethal concentration of *n*-butanol. We profiled, in triplicate, the growth characteristics and phospholipidomes of 116 different genetic constructs of *E. coli*, both in the presence and absence of 0.5% *n*-butanol (*v*/*v*). This led to the identification of 230 lipid species and subsequently to the reconstruction of the network of metabolites, enzymes and lipid properties driving the homeostasis of the *E. coli* lipidome. We were able to identify key lipids and biochemical pathways leading to altered *n*-butanol tolerance. The data led to new conceptual insights into the bacterial lipid metabolism which are discussed.

## 1. Introduction

The biosynthesis of *n*-butanol has received much attention over the past decades because of its potential in many different processes, most particular as a biofuel [1,2,3]. Chemical advantages of *n*-butanol over other biofuels are the high energy content and low hygroscopic- and corrosive properties. The lower flashpoint compared to diesel and ethanol makes *n*-butanol safer to work with. Additionally, with regard to human- and environmental health, *n*-butanol represents strongly reduced hazards and this contributes further to its interest [4,5,6]. Except as a biofuel, *n*-butanol has many other applications in chemistry such as in the production of paints, resins and plastics [7,8].

Bacterial strains of *Clostridium* have long been the model organisms for *n*-butanol production via acetone-butanol-ethanol fermentation (reviewed in [9]). Unfortunately, yields have remained rather low despite metabolic re-engineering and applying advanced co-cultures of micro-organisms [9,10,11]. This has spiked interest in engineering other model organisms such as *Saccharomyces* and *Escherichia coli* to produce *n*-butanol because of their excellent genetic accessibility [12,13,14,15].

Recovery of *n*-butanol from the production environment remains problematic [16]. Distillation is not energy efficient and hence not economically feasible. Adsorption of dissolved *n*-butanol with various sorbents has proven to be a very efficient process but unfortunately, desorption is often an issue, as is recycling of the sorbent [17]. Another technique that is often used to recover *n*-butanol, is gas stripping. Although this requires only moderate investments in machinery or materials, the excessive amounts of foam that are produced, have prevented a breakthrough for this method. Finally, also the performance of filters has been poor due to viscosity of *n*-butanol as an extractant [18]. Nevertheless, the efficiency of all methods increases with increasing *n*-butanol concentrations and achieving higher tolerance to *n*-butanol, remains key.

The intrinsic chemical properties of *n*-butanol present challenges to efficient bioproduction. *n*-butanol is a solvent in which hydrophobic compounds such as membrane lipids, readily dissolve. It is, therefore, self-evident that optimizing lipid metabolism can result in increased *n*-butanol tolerance. The high maximum solubility of *n*-butanol at room temperature (73 g/L or 7.7% *v*/*v*) makes it an ambitious goal to produce *n*-butanol at levels that would result in spontaneous phase separation. However, butanol-tolerant bacteria that can grow at this concentration have been isolated from the environment, demonstrating that this goal is realistic [19].

Toxic effects of organic solvents are due to detrimental effects on phospholipid membrane structure. Toxicity and hydrophobicity of primary alcohols are directly related [20,21]. Recent work has unraveled the different mechanisms by which membrane disturbance occurs [22]. These authors demonstrate how increasing *n*-butanol concentrations induce increased lipid disordering, hence increase membrane fluidity and permeability. However, the work is mainly on artificial membranes of simplified lipid composition, ignoring any bacterial capacity to adapt to a changing environment.

Increased short-chain alcohol tolerance can be achieved in *E. coli* by engineering active transport of these product out of the cell [23]. Intrinsically, genome wide generic stress responses are involved in improved *n*-butanol tolerance which include lipid membrane functions [24,25,26]. Involvement of lipid metabolism in response to *n*-butanol induced stress has been demonstrated in *Clostridia* [27], but for *E. coli* similar data is missing.

Here we explore this bacterial potential to adapt the lipidome in response to *n*-butanol exposure. We found a 50% inhibition of growth rate at 0.5% *n*-butanol (*v*/*v*) and chose to explore changes in lipid metabolism at this concentration. To this end, we overexpress all known lipid-related enzymes individually or, when possible, knock-out these genes. We thus aim to identify the pivotal genes of control and to identify marker lipids. Simultaneous recording of growth rates and biomass formation as characteristics of bacterial health link lipidome to *n*-butanol tolerance. This work thereby contributes to a rationale for optimization strategies in bioproduction of *n*-butanol and other hydrophobic compounds.

## 2. Results

**Figure 1:**
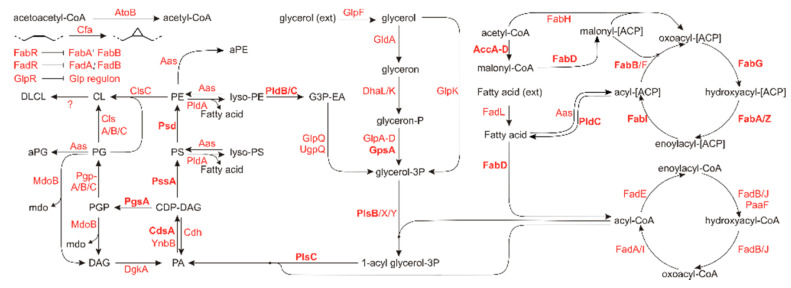
Metabolic scheme of lipid related genes and their role in lipid homeostasis. Genes essential for cell survival and multiplication under generic conditions are shown in bold. Adapted from [29]. Lipid abbreviations: aPE: N-acyl-phosphatidylethanolamine; aPG: O-acyl phosphatidylglycerol (headgroup acylated PG); CDP-DAG: cytidine diphosphate diacylglycerol; CL: cardiolipin; DAG: diacylglycerol; DLCL: di-lyso cardiolipin; PA: phosphatidic acid; PE: phosphatidylethanolamine; PG: phosphatidylglycerol; PGP: phosphatidylglycerolphosphate; PS: phosphatidylserine.

In *E. coli*, the phospholipidome is controlled by a relatively modest (known) set of 68 proteins of which only 20 are essential (as defined by the Keio collection [28]). These proteins are organized in several pathways related to fatty acid synthesis or -breakdown, glycerol backbone metabolism or are related to phospholipid modifications or -synthesis. These pathways and corresponding enzymes are visualized in Figure 1, highlighting the essential enzymes.

Cultures of strains overexpressing each of these genes individually were analyzed for growth characteristics (maximum growth rate and OD600 at steady state), as were the individual knock-outs of these genes. This allowed us to investigate lipidomes under maximum and minimum expression levels of lipid related genes.

### 2.1. Simplification of the Lipid Extraction Allows for More Detailed Lipidome Analysis

**Figure 2:**
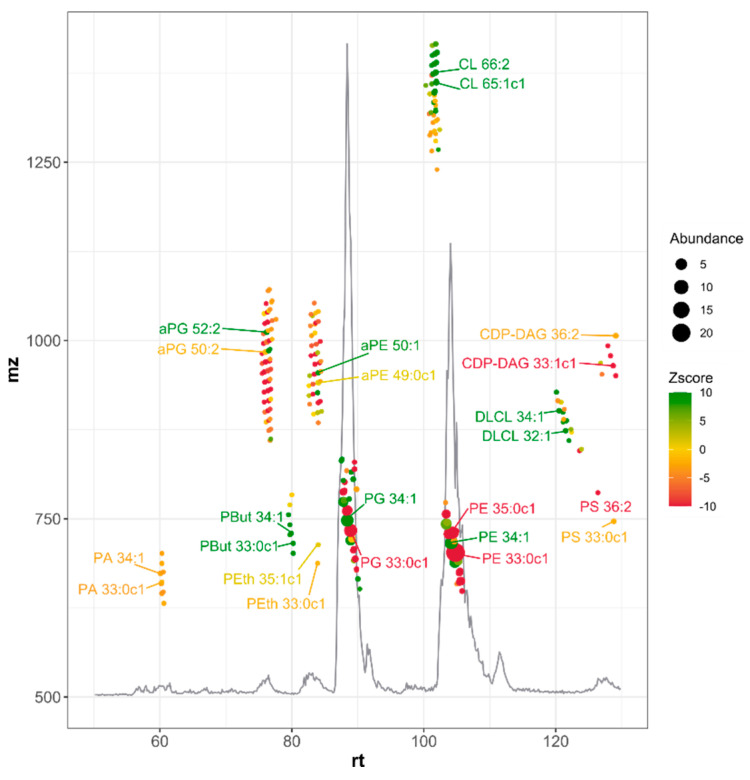
Base peak chromatogram of HILIC-based LC-MS analysis of wild type *E. coli* lipid extract. Lipid species are depicted, and color coded according to the Z-score of culturing in the presence of 0.5% *n*-butanol (green corresponding to higher abundance at *n*-butanol exposure). Dot size corresponds to abundance in WT *n*-butanol-free cultures. The two most abundant species of each lipid class are labeled. All lipid ions corresponded to deprotonated molecules. Examples of structures for each class of lipid ions are given in Supplementary Figure S1.

The simplified, one step extraction of lipids from the bacterial membranes resulted in a more complex lipidome than generally reported with liquid–liquid extraction [30,31] (Figure 2). This is in part due to the preservation of very polar lipids like CDP-DAG that normally partition towards the polar liquid phase in liquid–liquid extractions and are hence lost from the lipid extract. Simultaneously, the wide range of conditions analyzed here included conditions in which minor lipids became sufficiently abundant to survive noise filtering of raw data and were included. Forced integration of corresponding mz/rt features in samples where these compounds were initially not detected, assured reliable data for these minor compounds (e.g., aPE, aPG, DLCL) in all samples. In total, 230 distinct lipid species, distributed over 11 phospholipid classes were included in further analysis. Each lipid-related gene was individually overexpressed, and three separate cultures of the construct were extracted and analyzed. Similarly, knock-outs of each non-essential lipid-related gene were cultured in triplicate and extracted. As controls for the overexpressors and knock-out constructs, we used the *E. coli* wild-type strain (BW25113) with or without an empty overexpression vector, respectively. These controls were included on every 96-well plate used for culturing bacteria and were, therefore, conducted more than three times. In the very few cases where bacterial growth was insufficient to reach an optical density (OD600 nm) of 0.2, samples were excluded from further analysis. This resulted in the acquisition of 730 lipidomes (Table S1).

### 2.2. Random Forest Analysis Identifies Key Lipids and Lipid Genes

Breiman’s random forest analysis was applied to identify the key features [32]. Here, we applied random forest analysis to find lipid species that could classify the genetic status (gene ID and KO or OV) and *n*-butanol exposure (Yes/No) of cultured bacteria. Random Forest Analysis produced a top-10 panel of lipid species that collectively were able to perform accurate classification of all samples (Table 1).

Within the dataset, these lipids contribute only 21.2% of the total lipid related MS signal of wild-type bacteria. The top-4 species in Table 1 are within the 12 most abundant species in the total lipidome (Table S1). Notably, the other six species in Table 1 (PG 31:0c1 through aPE 50:1) are quantitatively minor compounds and consist nearly exclusively of phospholipid classes that are thus far mostly ignored in *E. coli* lipidomic papers, and their identification as marker lipid species was unexpected. The phospholipid class phosphatidylbutanol (PBut) is not a common class and its presence under standard growth conditions has not been reported. Formation of this lipid class is dependent on the presence of *n*-butanol in the medium. *n*-butanol is not a common constituent of growth medium nor is it a metabolite in the common metabolism of *E. coli*. However, we have previously shown that the cardiolipin synthase ClsB functions as a promiscuous phospholipase D, capable of exchanging phospholipid headgroups with primary alcohols [33]. In this light, it is not surprising to find highest levels of the PBut marker lipid in the ClsB overexpressing strain (Table 1).

**Figure 3:**
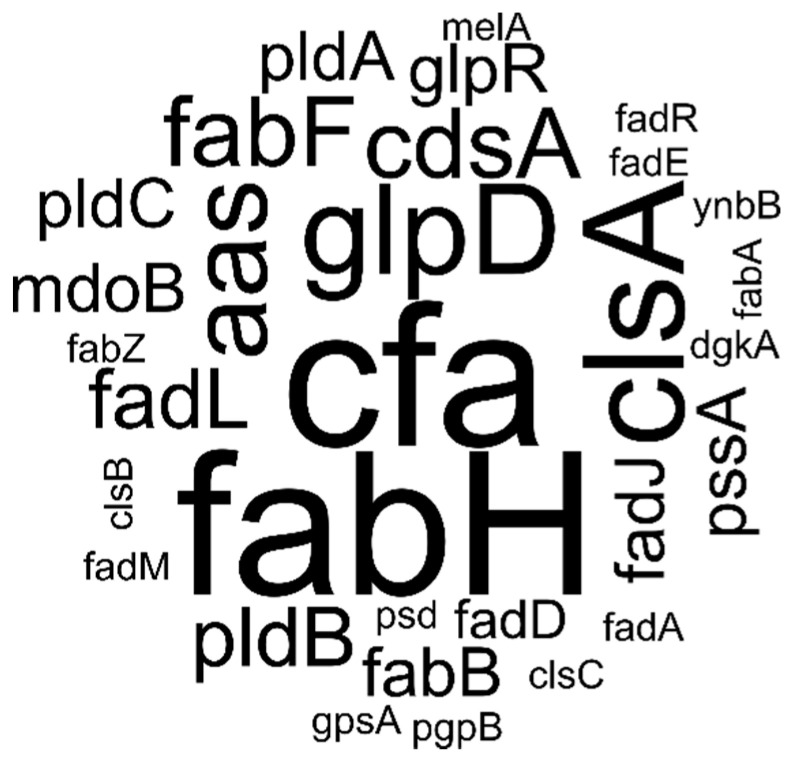
Tag cloud of the altered gene expression levels leading to extremes in marker lipid species identified by random forest analysis.

A less obvious but noteworthy characteristic of the lipid markers in Table 1 is the fact that every species has a distinct fatty acyl composition. It was reported previously that lipid species from distinct lipid classes but with the same acyl composition, displayed similar responses to genetic- and environmental challenges [29]. The presence of only distinct acyl compositions in the set of marker lipid species may thus reflect the random forest analysis algorithm eliminating information redundancy. Although it is apparent that the genes involved in achieving the extremes (Table 1) is not a random cross-section of all lipid related genes, care must be taken when interpreting this list. Particularly when looking at low lipid abundances, the gene expression modification leading to the lowest values, did often not differ significantly from the second or third lowest values. For instance, the knock-out of Cfa (the enzyme synthesizing the cyclopropane moiety) led to very low abundances of all marker lipid species containing a cyclopropane ring (indicated by the subscript ‘c1′ in the lipid name). To create a visual impression of the genes with highest impact on the marker lipids, we constructed a tag cloud from the 100 gene tags of the top- and bottom five genes in the 10 marker lipids (Figure 3). From the tag cloud it became evident that Cfa indeed has a large impact on the abundance of the marker lipid species. Other notable enzymes include FabH (involved in the initiation of fatty acid synthesis), ClsA (the dominant cardiolipin synthase), GlpD (glycerol backbone synthesis) and Aas (headgroup acylation).

### 2.3. The Lipidome Is Organized in Clusters of Closely Connected Lipid Species

**Figure 4:**
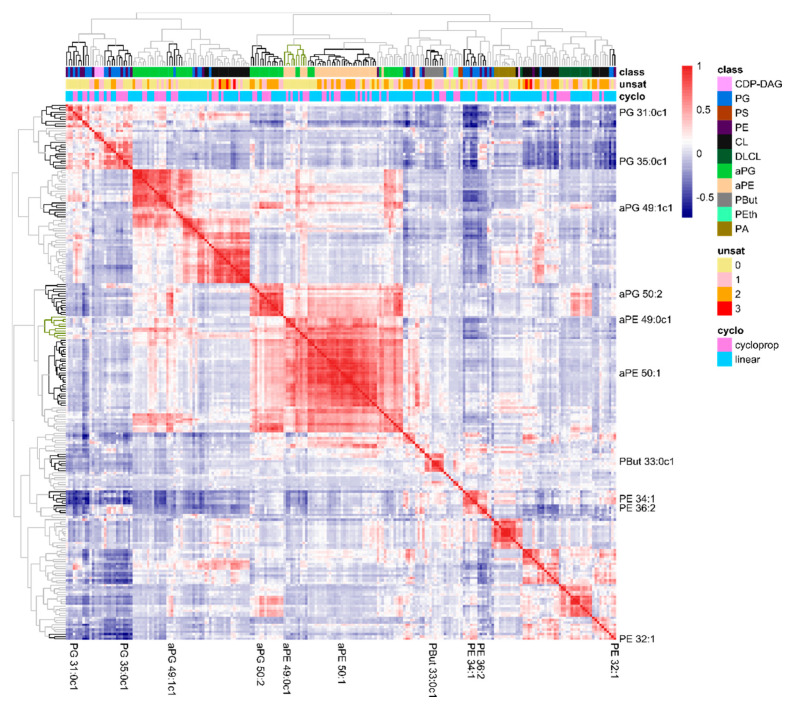
Heatmap of correlations in the lipidome composition of all 700+ samples. The position of the ten marker lipids is indicated and the clusters are emphasized in the hierarchical trees (**top** and **left**). The colored bars (**top**) indicate the phospholipid class off each lipid species, the total number of unsaturations in the acyl chains and whether the acyl chains are linear of contain a cyclopropane moiety.

Next, we investigated the underlying concepts that defined these ten lipids as informative markers. We hypothesized that these markers were representatives of clusters of lipid species defining functional domains in lipid membranes, or at least of clusters of lipid species whose abundances in the membrane are closely related. Therefore, we constructed a heatmap of lipid–lipid correlations and organized lipids by hierarchical clustering (Figure 4). Indeed, clusters of lipids with strongly correlating abundances (dark blue or -red) were visible, correlating lipids within one cluster (on the diagonal) or between different clusters (off-diagonal). Notably, the marker lipids from Table 1 were scattered over these clusters. Within clusters, only a single marker lipid was present. As an exception, the second most abundant lipid species in *E. coli*, PE 32:1 and part of the identified marker lipids, appeared not to be part of any lipid cluster (bottom right corner). The horizontal color bars on top of the heatmap, visualize lipid class, degree of unsaturation and presence of cyclopropane rings of the individual lipids within the clusters. Although we detected clusters of lipids from the same lipid class (particularly for the headgroup acylated lipid classes aPE and aPG), all major lipid classes were divided over several clusters. Similarly, lipid unsaturation or the presence of cyclopropane moieties were not driving forces of the lipid clusters formation. Hence, the formation of lipid clusters in the correlation heatmap was a result of a complex interplay of multiple processes.

### 2.4. Validation of Metabolic Pivot Points

We observed large differences between minimum and maximum contributions of the marker lipids to the lipidome (Table 1). Even the abundance of major compounds can be manipulated to fall below 1% without loss of viability. PE 32:1 the observed range in abundancies was particularly wide (0.75–24.82%). Notably, these extremes are both obtained by alteration of the expression level of the *cfa* gene. Although PE 32:1 itself does not contain a cyclopropane ring, our data point towards an important role for PE 32:1 as a substrate for the Cfa enzyme. Lack of this enzyme leads to extensive accumulation of this lipid species, whereas overexpression resulted in nearly complete depletion. Concomitantly, the abundance of PE 33:0c1 (the Cfa product of PE 32:1) fell under these conditions from 25.9 ± 0.5% (Cfa overexpression) to 1.5 ± 0.2% (Cfa knock-out; Table S1). The lipid species PE 34:1, which also contains one single unsaturation that may be targeted by Cfa, was much less affected by Cfa overexpression than PE 32:1. This further illustrates the complex control of the phospholipidome.

**Figure 5:**
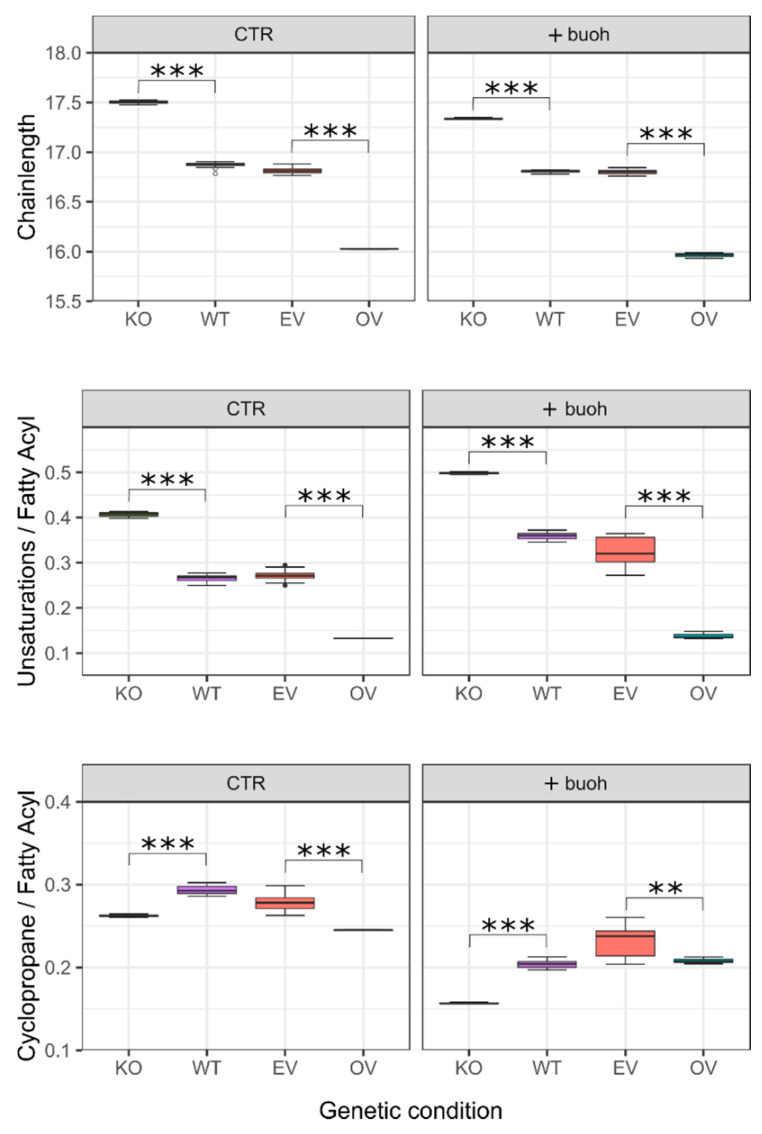
Boxplots demonstrating the effect of alteration of FabH levels on key properties of membrane lipids. KO and OV represent gene knock-out and overexpression, respectively. EV represents the wild-type (WT) transformed with an empty overexpression vector. Panels on the right (+buoh) were obtained from cells grown under identical conditions as the control (CTR) but in the presence of 0.5% (*v*/*v*) *n*-butanol. It should be noted that acyl properties were calculated from the data in supplementary Table S1 and that the values at y-axis should not be treated as absolute. A discrepancy may result from the fact that differences in ionization efficiency can exist between molecular species. Differences between the two control conditions (wild type WT and empty vector EV) were non-significant in all experiments shown. Statistical significances (based on uncorrected *p*-values) of KO and OV are shown with respect to their controls (WT and EV, respectively). **: *p* < 0.01; ***: *p* < 0.001.

The high impact FabH has on the marker lipids (Figure 3), was investigated in more detail. FabH is involved in the initiation of fatty acid synthesis by coupling acetyl-CoA to malonyl-ACP, generating an oxoacyl-ACP and recycling free coenzyme-A. After a series of reductions by other Fab-proteins, the newly synthesized C4-fatty acyl-ACP is elongated using a new molecule of acetyl-CoA and this process typically continues until a chainlength of C14 or longer is achieved. Increasing expression of FabH (KO < (WT or EV) < OV) resulted in a decrease of average fatty acyl chainlength from 17.5 to 16.0 carbon atoms (Figure 5, top row). This means that bilayer thickness may be expected to decrease by 10% upon FabH overexpression. The presence of *n*-butanol in the culture medium did not affect this trend, although it should be mentioned that in the knock-out, the decrease in average chainlength from 17.5 (CTR) to 17.3 (+butanol) was statistically significant (*p* < 0.01). It is, however, unlikely to influence biophysical properties of the membrane.

The degree of unsaturation of lipid acyl chains was affected in a similar way as the chainlength. The average number of unsaturations per fatty acyl chain dropped from 0.41 in the KO to 0.27 in the combined WT and EV samples (*p* < 5 × 10^−5^), and again significantly further to 0.13 in the OV samples (*p* < 2 × 10^−16^). Hence, the FabH overexpressor has only 31% of the unsaturations the knock-out has and this may be expected to reduce membrane fluidity. Cyclopropane moieties are synthesized from unsaturated fatty acids and abundance of these characteristics are overall negatively correlated. However, the amount of cyclopropane containing fatty acids was not nearly affected to the extend observed for chainlength and unsaturation, but culturing in the presence of *n*-butanol consistently reduced the presence of cyclopropane containing fatty acids (Figure 5, bottom row).

### 2.5. Linking Lipid Related Genes to n-Butanol Tolerance

The toxic properties of *n*-butanol lead to an approximate 50% decrease in growth rate for WT *E. coli*. We investigated the effect of gene expression alterations on both the specific growth rate as well as on the OD600 achieved in steady state, the latter being a measure for biomass. Bacterial strains with higher metabolic efficiency are able to use more nutrients for cell growth and division but this not necessarily corresponds to higher growth rates.

**Figure 6:**
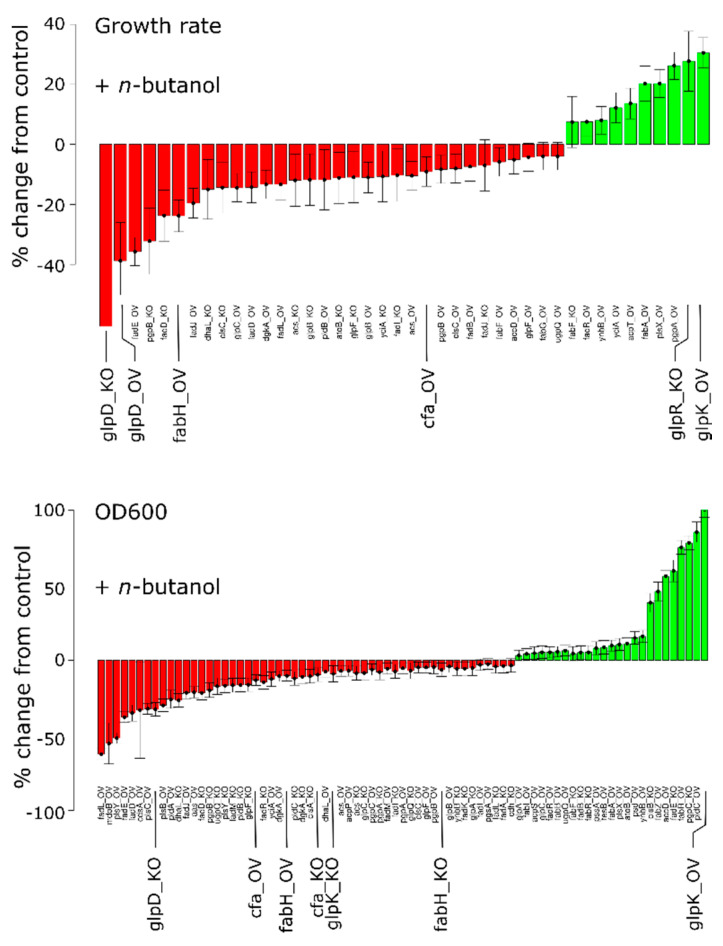
Specific growth rates and optical densities at steady state. Values of genetic constructs are compared to controls. Only data that were statistically significant (*p* < 0.05) are shown. Genes discussed in the text are indicated by larger font size.

When grown in the presence of *n*-butanol, 42 out of the 116 genetic constructs had maximum specific growth rates distinct from controls (Figure 6 and Supplementary Table S3 for all growth data). Notably, in ten of these the growth rate was higher, suggesting that the extreme genetic conditions in our OV/KO strains cannot functionally be achieved in the wild-type strain by regulation of gene expression, translation and/or enzyme activity. Looking at these extremes, it stands out that alterations in glycerol metabolism have very pronounced effects. Highest growth rates were achieved by overexpressing the (glycerol) kinase GlpK or knocking-out the Glp repressor GlpR. Lowest growth rates on the other hand, were achieved by either knocking-out or overexpressing GlpD, the only aerobic variant of the four glycerol-3-phosphate dehydrogenases [34]. Fatty acid characteristics for gene constructs involving GlpD and GlpK are summarized in Supplementary Figure S2.

The optical density (i.e., biomass) achieved at steady state was more variable between growth conditions than the growth rate (Figure 6, bottom panel). Most conditions did have a biomass production that was different from control conditions. Interestingly, GlpK overexpression stimulated growth rate as well as biomass production, whereas GlpD knockout on the other hand, impaired both growth rate and biomass production. Both enzymes are involved in both lipid metabolism and central carbon metabolism but from these data it is impossible to determine which of these metabolic pathways is responsible for the altered growth characteristics.

## 3. Discussion

As a powerful machine learning algorithm, if applied to properly generated data sets, Breiman’s random forest analysis can generate robust results that are applicable to previously unseen data. It is essential that the data sets are created in a steady fashion with reliable normalization standards [35]. The current setup, where bacteria were grown in multiwell plates and lipid extractions was simplified to a one-step procedure, enabled us to generate such a large training set. Since a lipidome can be analyzed within five minutes (Figure 2), the collective samples could be processed within only three days of LC-MS analysis. The resulting set of ten marker lipids not only could be used to identify the genetic status and *n*-butanol exposure of all samples but also helped in identifying clusters of functionally related lipids. The exact nature of how lipids within these lipid clusters are related is not yet fully understood but clearly this exceeds just lipid class or acyl composition. Particularly abundant or sparse lipid clusters can be correlated to specific genetic conditions, and this can provide further leads for investigation.

It should be noted that distinct cultures of the same genetic construct of *E. coli*, produced highly consistent lipidome compositions. Therefore, statistical significance was easily obtained between different strains or conditions, even when changes were too small to be expected to alter biophysical properties of the membranes. Put differently: statistical significance should not be confused with biological relevance and we have tried to highlight relevant processes rather than to report as many differences as possible. Readers are encouraged to query the dataset for changes in processes of their own interests.

Because of the experimental setup in which half of the samples were cultured in the presence of *n*-butanol, it can be expected that many of the phenomena we observed were related to coping with induced membrane stress. We expect this to be reflected in the composition of the ten marker lipids. Indeed, most of the marker lipids will have high impact on lipid packing in the membrane. This is particularly the case for the headgroup acylated lipid species aPE and aPG, where the additional acyl chain will fold back into the hydrophobic region of the bilayer, thus profoundly changing the shape of the phospholipid and increasing the inter- and intra-molecular hydrophobic interactions. In fact, membrane splaying as a driving force for membrane collapse (i.e., disturbing lipid–lipid interactions by accumulation of *n*-butanol at the phospholipid headgroups [22]), may be effectively counteracted. In this respect, it is important to acknowledge that these headgroup acylates have mostly been ignored in *E. coli* lipidomic papers due to low abundance and/or unfamiliarity of scientists with this lipid class. It can be very worthwhile to reassess results of these papers given these new insights and to consider re-analyzing crucial experiments. Our rapid and labor-extensive lipid extraction and short analysis time will aid in making such goals achievable.

Although the HILIC-based lipidomic workflow enabled us to note and identify unusual lipid classes, we were only able to identify lipids at the species level, i.e., data lack information about acyl composition and -position. When a more detailed level of information is essential, modern very fast MS/MS instruments may allow profiling at the fatty acyl (position) level. Alternatively, researchers may opt for reverse-phase approaches where separation is primarily dictated by the acyl composition. However, better species resolution comes at the cost of longer analysis times [36].

Cyclopropane moieties are synthesized by the enzyme Cfa from an unsaturation in an acyl chain. The measure of unsaturated acyl chains in a biomembrane is typically considered to be the key property when assessing fluidity of membranes. Since there exists no ‘saturase’ that will remove a double bond from a fatty acyl, converting a double bond to a rigid cyclopropane moiety will be an effective way to counteract the increased membrane fluidity induced by *n*-butanol partitioning [37]. Indeed, the role of cyclopropane fatty acids in organic solvent tolerance was demonstrate in *Pseudomonas putida* [38] and also in other bacteria including *E. coli*, a role in coping with environmental stress has firmly been established [39,40,41]. In a recent paper, we have shown that, even compared to any other lipid-related gene, both deletion and overexpression of Cfa has very profound effects on the overall lipidome of *E. coli* [29]. It is, therefore, not surprising that Cfa was among the most relevant genes in Figure 3. However, the *cfa* knockout did not have a reduced growth rate. Additionally, biomass increase during growth was only marginally lower than WT, indicating that Cfa has limited importance in dealing with butanol stress.

Growth data pointed towards a crucial role for glycerol metabolism rather than fatty acid- or phospholipid metabolism in *n*-butanol tolerance. Overexpression of GlpK led to the highest growth rate as well as a large increase in biomass. GlpK is a known point of (allosteric) regulation of carbon metabolism [42,43] and has been linked to cell stress response [44]. Furthermore, substrate specificity is not particularly high, which makes GlpK active in several metabolic pathways [45]. The effect of GlpK overexpression may therefore reflect its involvement in responses unrelated to lipid metabolism. The lack of a clearly aberrant lipidome in this construct supports this idea.

Manipulation of GlpD levels on the other hand, had strong negative effects on both growth rate and biomass. GlpD is a key enzyme in glycerol metabolism as it was shown that growth on glycerol strictly depends on the presence of GlpD [46]. Hence, the availability of physiological levels of the phospholipid precursor glycerol-3P appears to be an important prerequisite for a healthy lipid homeostasis.

Taken together, this large-scale MS-based lipidomic approach has revealed the presence of clusters of lipids whose abundance is related. It was also demonstrated that the 116 different genetic constructs and growth conditions can be classified by ten marker lipids. The abundance of these marker lipids was shown to be highly variable. Furthermore, these data show a strong link between lipid related genes and *n*-butanol tolerance. This opens new perspectives for targeted membrane engineering in achieving higher membrane resistance to organic solvents.

## 4. Materials and Methods

### 4.1. Chemicals

Isopropyl β-D-1-thiogalactopyranoside (IPTG) was obtained from Melford (Suffolk, UK). NaCl, *n*-butanol, and formic acid and were purchased from Merck Chemicals (Darmstad, Germany). Chloramphenicol was obtained from Boehringer Mannheim (Mannheim, Germany). Chemicals were of the highest purity available. Trypton, agar, and kanamycin were purchased from Sigma (St. Louis, MO, USA). Yeast extract was obtained from MP Biomedicals (Strasbourg, France). Methanol, acetonitrile (ACN), acetone and ammonium formate were purchased from BioSolve (Valkenswaard, The Netherlands), chloroform was obtained from Roth (Karlsruhe, Germany) and were all HPLC/MS grade.

### 4.2. Bacterial Strains, Growth Conditions and Plasmids

The bacterial strains and plasmids used in the study are listed in Supplementary Materials and Methods (Supplementary Table S2). *E. coli* strain BW25113 and its derivates are part of the Keio collection [28], obtained from NBRP (NIG, Mishima, Japan). *E. coli* strains were routinously grown at 37 °C in Luria Bertani (LB) broth or on LB agar plates. Plasmids overexpressing lipid genes present in *E. coli* strain AG1(ME5305) (part of the ASKA(-) collection [47] obtained from NBRP (NIG, Mishima, Japan) were transferred to *E. coli* strain BW25113. When appropriate, growth media were supplemented with chloramphenicol (34 μg/mL) or kanamycin (50 μg/mL).

Protein expression was induced with a final concentration of 10 μM IPTG for the ASKA(-) clones since the ORF on the pCA24N plasmid are under control of IPTG-inducible promoter, P_T5-*lac*_. For phospholipid analysis, cultures (150 μL) were grown in 96 wells plates, in a Versamax microtiter plate reader (Molecular Devices, Sunnyvale, CA, USA), in the absence or presence of 0.5% (*v*/*v*) *n*-butanol. To this end, pre-cultures that had research stationary phase in 9 h growth, were diluted 1:100 and growth was followed. The plate was covered using a clear film to reduce evaporation. Absorbance at 600 nm was measured every 5 min. These data were used to calculate maximum specific growth rates as defined by the slope of a linear fit to log transformed OD values.

### 4.3. Lipid Extraction

Lipids were extracted as described before [29]. In brief, bacterial cultures were transferred to glass coated plates, centrifuged (1800× *g*, 20 min, 4 °C) and the medium was completely removed by aspiration. Pellets were resuspended without further washing in 150 µL chloroform/methanol (1:1 *v*/*v*), extracted for 1 h at 4 °C, followed by centrifugation (1800× *g*, 20 min, 4 °C). The extracts were transferred to a new glass coated 96 well plate that was covered by a thin sheet of aluminum foil and placed in the autosampler. WT strain, empty vector, and cell free incubations were included with every plate. Cell free incubations did not produce meaningful lipid signals and these samples were excluded from further analysis.

### 4.4. Liquid Chromatography Mass Spectrometry of Lipids

LC-MS of phospholipids was performed as described before [29,48]. In brief, 10 µL of the supernatant was injected onto a hydrophilic interaction liquid chromatography (HILIC) column (2.6 μm HILIC 100 Å, 50 × 4.6 mm, Phenomenex, Torrance, CA, USA), and eluted with a gradient from ACN/Acetone (9:1, *v*/*v*) to ACN/H_2_O (7:3, *v*/*v*) with 10 mM ammonium formate, and both with 0.1% formic acid at a flow rate of 1 mL/min. Gradient development was as follows (time in min, %B): (0, 0), (1, 50), (3, 50), (3.1, 100), (4, 100). Regeneration time between subsequent runs was 55 s, which was identical to our autosampler injection time and therefore not listed separately. The column outlet of the LC was connected to a heated electrospray ionization (HESI) source of a LTQ XL mass spectrometer (ThermoFisher Scientific, Waltham, MA, USA). Full scan spectra were collected in negative ionization mode in the range from 350–1750 amu at a scan speed of 3 scans/s. Source- and capillary temperatures were set to 450 and 400 °C, respectively, and the ionization voltage to −2.5 kV. Calibration curves with constructed using authentic standards of PE, PG and CL to ensure linear responses for the lipid concentrations used in the experiments. Due to the lack of standard for all lipid classes (e.g., phosphatidylbutanol, head group acylated PG), we have refrained from conversion of lipid data to absolute quantities.

### 4.5. Data Analysis

LC-MS data were converted to mz(X)ML format using MSconvert and analyzed using XCMS version 1.52.0 using R version 3.4.4 (15 March 2018) [49,50,51]. Phospholipid (sub-)classes were identified based on retention time and mz/rt features were matched against an in-silico generated lipid database. Lipid de-isotoping was conducted using a natural abundance of ^13^C of 1.1%. Lipids were reported on the lipid species level rather than lipid class level mass based on the well documented absence of ether lipids in *E. coli* [52]. A normalization was performed such that the sum of all species is 100% in every sample. An overall cross-comparison on lipidomes was performed by using the CyberT tool package [53]. BayesAnova tests were applied on all possible lipidome combinations (parameters: sliding window size 101, Bayes confidence estimate value 5, and PPDE post processing treatment). Comparison resulted with an adjusted *p*-value lower than 0.05 were considered significant. Breiman’s random forest analysis (500 trees and 100 iteration) was applied to the normalized data set by using Weka 3.9 [54]. For significant feature selection, sequential backward selection was used with the 34OOB error minimization option. During the analysis, 10-fold cross-validation was applied. The feature importance evaluations were sorted by eliminating the least important features.

## Figures and Tables

**Table 1:** Most informative lipid species for the classification of genetic composition and *n*-butanol exposure (yes/no) as identified by random forest analysis. Percentages are given as contributions to the total mass spectrometry signal.

Lipid Species	Average (%)	Max. (%)	Min. (%)	Genes ^1^ (Extremes)
PE 34:1	7.67	17.41	0.72	*glpD* (OV); *fabH* (OV)
PE 32:1	5.75	24.82	0.72	*cfa* (KO); *cfa* (OV)
PE 36:2	4.20	12.09	0.84	*fabH* (KO); *fabH* (OV)
PG 35:0c1	2.60	10.47	0.20	*glpR* (KO); *aas* (OV)
PG 31:0c1	0.35	1.21	0.05	*fabH* (OV); *fabH* (KO)
aPG 50:2	0.26	2.94	0.03	*fadL* (OV); *fabF* (OV)
aPE 49:0c1	0.15	0.91	0.01	*aas* (OV); *cfa* (KO)
aPG 49:1c1	0.10	1.00	0.01	*pldA* (OV); *fabH* (KO)
PBut 33:0c1	0.07	2.99	0.00	*clsB* (OV); *fadD* (KO)
aPE 50:1	0.07	1.34	0.00	*aas* (OV); *clsA* (OV)

^1^ The genes of which altered expression resulted in highest or lowest abundance are given.

## Data Availability

Lipidomic peak lists and growth data of individual bacterial cultures have been made available as supplementary data.

## Supplementary Materials

The following are available online at https://www.mdpi.com/article/10.3390/metabo11050286/s1, Table S1: Normalized lipidomic analysis of bacterial constructs. Table S2: bacterial strains and plasmids. Figure S1: Lipid class structures. Figure S2: lipid characteristics for GlpD and GlpK.
