# Supplementary material for: Control of n-Butanol Induced Lipidome Adaptations in E. coli
Source: Metabolites. 2021 Apr 29;11(5):286. doi: 10.3390/metabo11050286 (PMC8145963; doi:10.3390/metabo11050286)
Supplement: Supplementary file 1 [file metabolites-11-00286-s001.zip › Supplementary Figure S1 Structures of lipid classes.pdf]

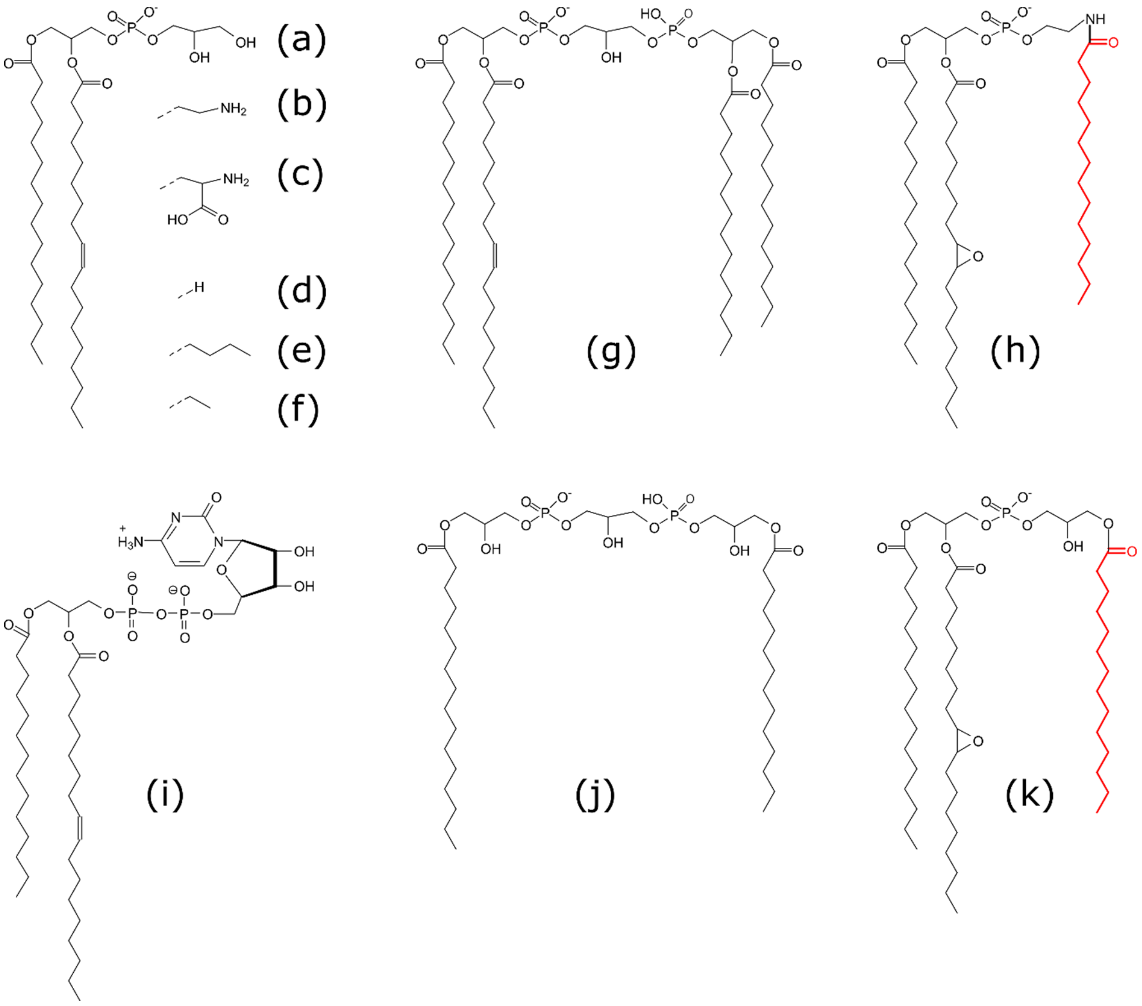

Figure S1 Structures of lipid classes described in this paper. All lipids are depicted as deprotonated molecules. High resolution accurate mass fragmentation spectra have been published previously (e.g. https://doi.org/10.1016/j.celrep.2019.04.018). (a) PG; (b) PE; (c) PS; (d) PA; (e) PBut; (f) Peth; (g) CL; (h) aPE; (i) CDP-DAG; (j) DLCL; (k) aPG. Headgoup acylations are depicted in red. Note the cyclopropane moieties in (h) and (k).
