# Supplementary material for: Control of n-Butanol Induced Lipidome Adaptations in E. coli
Source: Metabolites. 2021 Apr 29;11(5):286. doi: 10.3390/metabo11050286 (PMC8145963; doi:10.3390/metabo11050286)
Supplement: Supplementary file 1 [file metabolites-11-00286-s001.zip › Supplementary Figure S2 GlpD and GlpK effects.pdf]

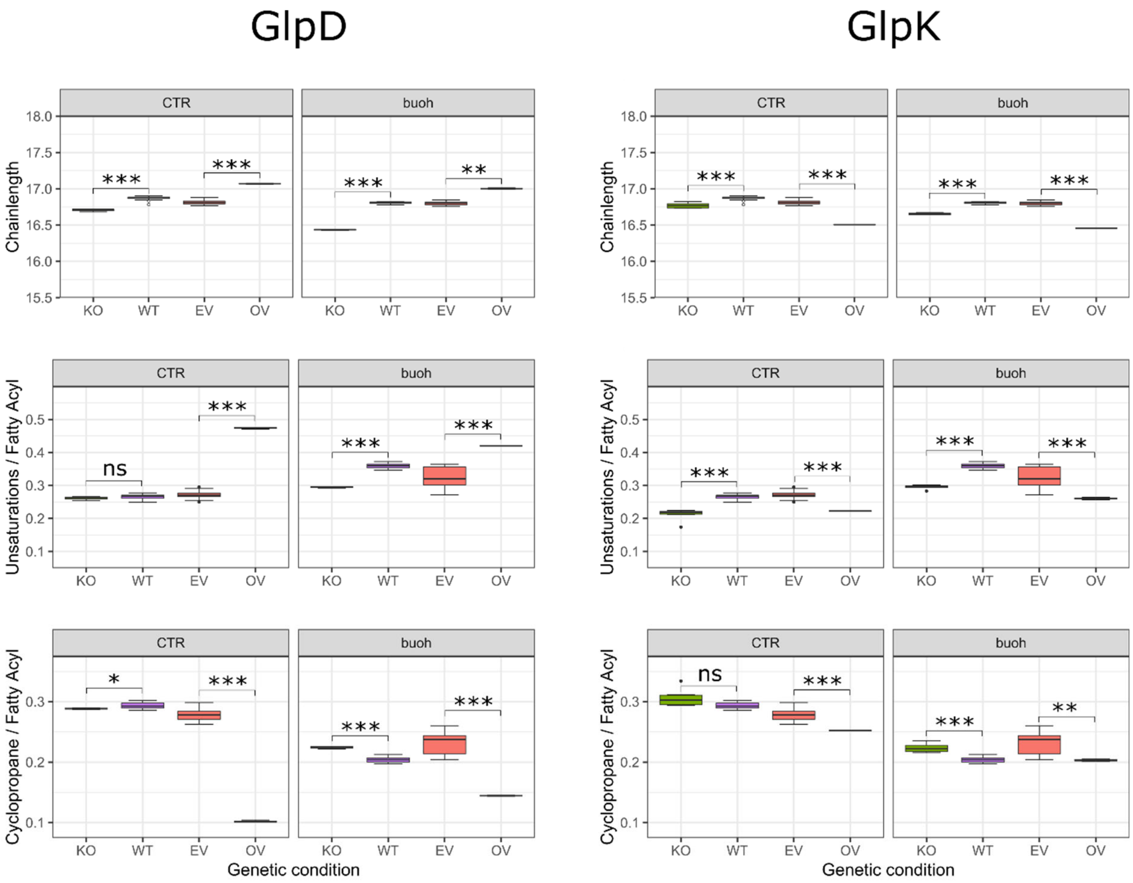

Supplementary Figure S2: Boxplots demonstrating the effect of alteration of GlpD (left) or GlpK (right) levels on key properties of membrane lipids. KO and OV represent gene knock-out and overexpression, respectively. EV represents the wild-type (WT) transformed with an empty overexpression vector. Panels on the right (+ buoh) were obtained from cells grown under identical conditions as the control (CTR) but in the presence of 0.5% (v/v) *n*-butanol. It should be noted that acyl properties were calculated from the data in Supplementary Table S1 and that the values at y-axis should not be treated as absolute. A discrepancy may result from the fact that differences in ionization efficiency can exist between molecular species. Differences between the two control conditions (Wildtype WT and empty vector EV) were non-significant in all experiments shown. Statistical significances (based on uncorrected p-values) of KO and OV are shown with respect to their controls (WT and EV, respectively). NS: not significant, *: $p < 0.05$; **: $p < 0.01$; ***: $p < 0.001$.
